# Atrial fibrillation alters the microRNA expression profiles of the left atria of patients with mitral stenosis

**DOI:** 10.1186/1471-2261-14-10

**Published:** 2014-01-25

**Authors:** Hai Liu, Guang-xian Chen, Meng-ya Liang, Han Qin, Jian Rong, Jian-ping Yao, Zhong-kai Wu

**Affiliations:** 1 Second Department of Cardiac Surgery, First Affiliated Hospital of Sun Yat-Sen University, 58 Zhongshan II Road, Guangzhou 510080, China; 2 Department of Cardiopulmonary Bypass, First Affiliated Hospital of Sun Yat-Sen University, Guangzhou, China

**Keywords:** Atrial fibrillation, Microrna, Mitral stenosis, Microarray, Mirfocus

## Abstract

**Background:**

Structural changes of the left and right atria associated with atrial fibrillation (AF) in mitral stenosis (MS) patients are well known, and alterations in microRNA (miRNA) expression profiles of the right atria have also been investigated. However, miRNA changes in the left atria still require delineation. This study evaluated alterations in miRNA expression profiles of left atrial tissues from MS patients with AF relative to those with normal sinus rhythm (NSR).

**Methods:**

Sample tissues from left atrial appendages were obtained from 12 MS patients (6 with AF) during mitral valve replacement surgery. From these tissues, miRNA expression profiles were created and analyzed using a human miRNA microarray. Results were validated via reverse-transcription and quantitative PCR for 5 selected miRNAs. Potential miRNA targets were predicted and their functions and potential pathways analyzed via the miRFocus database.

**Results:**

The expression levels of 22 miRNAs differed between the AF and NSR groups. Relative to NSR patients, in those with AF the expression levels of 45% (10/22) of these miRNAs were significantly higher, while those of the balance (55%, 12/22) were significantly lower. Potential miRNA targets and molecular pathways were identified.

**Conclusions:**

AF alters the miRNA expression profiles of the left atria of MS patients. These findings may be useful for the biological understanding of AF in MS patients.

## Background

Atrial fibrillation (AF) is characterized as an irregular and sometimes rapid heart rate, with symptoms that include palpitations and shortness of breath. AF is the most common cardiac arrhythmia observed in clinical practice and constitutes a risk factor for ischemic stroke [1]. Despite recent significant advances in the understanding of the mechanisms associated with AF, complexities in the etiology of atrial electrical dysfunction (including a genetic component [2]) and the subsequent associated arrhythmia have prevented definitive elucidation [3].

The progression from acute to persistent and then chronic AF is accompanied by changes in gene expression that lead to differences in protein expression and activity. MicroRNAs (miRNAs) are regulators of gene expression at the post-transcriptional level [4], and appear to have regulatory roles that underlie the pathophysiology of AF. Many studies have shown that miRNAs regulate key genetic functions in cardiovascular biology and are crucial to the pathogenesis of cardiac diseases such as cardiac development [5], hypertrophy/heart failure [6], remodeling [7], acute myocardial infarction [8], and myocardial ischemia-reperfusion injury [9]. Currently, there is a growing body of literature that indicates that many miRNAs are involved in AF through their target genes [7,10,11].

AF can be an isolated condition, but it often occurs concomitantly with other cardiovascular diseases such as hypertension, congestive heart failure, coronary artery disease, and valvular heart disease [12]. AF is also prevalent in mitral stenosis (MS; a consequence of rheumatic fever), affecting approximately 40% of all MS patients [13]. MS is among the major cardiovascular diseases in developing countries where rheumatic fever is less well controlled, and 50% or more of patients with severe MS have AF. Patients with both AF and MS have a 17.5-fold greater risk of stroke and a four-fold higher incidence of embolism compared with people with normal sinus rhythm (NSR) [14,15].

Structural changes of the left atria (LA) and right atria (RA) associated with AF in MS patients are well established [13,14]. Recently, reports suggest that AF also alters the miRNA expression profiles in RA of MS patients [16,17]. However, miRNA changes in LA from MS patients with AF are still unknown. Given the complexity of the pathophysiology that may be associated with AF, we need a better understanding of the miRNA changes in the LA, which may help in designing and developing new therapeutic interventions. This study investigated alterations of miRNA expression profiles in LA tissues of MS patients with AF relative to MS patients with NSR.

## Methods

The Human Ethics Committee of First Affiliated Hospital of Sun Yat-sen University approved this study, and the investigation complied with the principles that govern the use of human tissues outlined in the Declaration of Helsinki. All patients gave informed consent before participating in the study.

### Human tissue preparation

Left atrial appendage (LAA) tissue samples were obtained from MS patients, both in NSR (n = 6, without history of AF) and with AF (n = 6, documented arrhythmia >6 months before surgery). The tissue samples were obtained at the time of mitral valve surgery, immediately snap frozen in liquid nitrogen, and stored at -80°C until used. The diagnosis of AF was reached by evaluating medical records and 12-lead electrocardiogram findings. NSR patients had no history of using antiarrhythmic drugs and were screened to ensure that they had never experienced AF [18]. Preoperative 2-dimensional color transthoracic echocardiography was performed routinely on the patients. Preoperative functional status was recorded according to New York Heart Association (NYHA) classifications.

### RNA isolation

The total RNA from human LAA tissue samples was extracted using TRIzol reagent (Invitrogen) in accordance with the protocol of the manufacturer. The RNA quality of each sample was determined using an Agilent 2100 Bioanalyzer (Agilent Technologies, Santa Clara, CA, USA) and immediately stored at -80°C.

### Microarray processing and analysis

The miRNA microarray expression analysis was performed by LC Sciences (Houston, TX, USA) as described previously [19]. In brief, the assay began with a total RNA sample (2 to 5 μg). The total RNA was size-fractionated using a YM-100 Microcon centrifugal filter (Millipore, Billerica, MA). RNA sequences of <300 nt were isolated. These small RNAs were then extended at the 3′ end with a poly(A) tail using poly(A) polymerase, and then by ligation of an oligonucleotide tag to the poly(A) tail for later fluorescent dye staining.

Hybridization was performed overnight on a μParaflo microfluidic chip using a micro-circulation pump (Atactic Technologies, Houston, TX). Each microfluidic chip contained detection probes and control probes. The detection probes were made *in situ* by photogenerated reagents. These probes consisted chemically of modified nucleotide coding sequences complementary to target miRNA (all 1921 human miRNAs listed in the Sanger’s miRNA miRBase, Release 18.0, http://microrna.sanger.ac.uk/sequences/) and a spacer segment of polyethylene glycol to extend the coding sequences away from the substrate. The hybridization melting temperatures were balanced by chemical modifications of the detection probes. Hybridization was performed using 100 μL of 6× saline-sodium phosphate-EDTA (SSPE) buffer (0.90 M NaCl, 60 mM Na_2_HPO4, 6 mM EDTA, pH 6.8) containing 25% formamide at 34°C.

Fluorescence labeling with tag-specific Cy5 dye was used for after-hybridization detection. An Axon GenePix 4000B Microarray Scanner (Molecular Device, Union City, CA) was used to collect the fluorescent images, which were digitized using Array-Pro image analysis software (Media Cybernetics, Bethesda, MD). Each miRNA was analyzed two times and the controls were repeated 4-16 times.

Analysis of the microarray data was also performed at LC Sciences (see Additional file 1). The microarray data was analyzed by subtracting the background, and then the signals were normalized using a locally weighted regression scatterplot smoothing (LOWESS) filter as reported previously [20]. Detectable miRNAs were selected based on the following criteria: signal intensity >3-fold the background standard deviation, and spot coefficient of variation (CV) < 0.5, where CV = standard deviation/signal intensity. When repeating probes were present on the array, the transcript was listed as detectable only if the signals from at least 50% of the repeating probes were above detection level. To identify miRNAs whose expression differed between the AF and NSR groups, statistical analysis was performed. The ratio of two samples was calculated and expressed in log_2_^scale (balanced)^ for each miRNA. The miRNAs were then sorted according to their differential ratios. The *P*-values of the *t*-test were also calculated. miRNAs with *P*-values < 0.05 were considered significantly differentially expressed.

### Reverse transcription-real time quantitative PCR (RT-qPCR) validation of selected miRNAs

To validate the microarray results in the present study, a stem-loop RT-qPCR based on SYBR Green I was performed on selected differentially expressed miRNAs. The primers used are listed in Additional file 2. Total RNA was isolated using TRIzol reagent (Invitrogen) as described above. A single-stranded cDNA for each specific miRNA was generated by reverse transcription (RT) of 250 ng of total RNA using a miRNA-specific stem-looped RT primer. Briefly, an RT reaction mixture contained 250 ng of total RNA, 0.5 μL of 2 μM stem-loop RT primer, 1.0 μL of 5× RT buffer, 0.25 μL of 10 mM of each dNTP, 0.25 μL of 40 U/μL RNase inhibitor, and 0.5 μL of 200 U/μL Moloney murine leukemia virus (M-MLV) reverse transcriptase. An Eppendorf Mastercycler (Eppendorf, Hamburg, Germany) was used to perform the RT reaction under the following conditions: 42°C for 60 min, 70°C for 15 min, and finally, held at 4°C.

After the RT reaction, qPCR was performed using an ABI PRISM 7900HT sequence-detection system (Applied Biosystems, Foster City, CA, USA) with the Platinum SYBR Green qPCR SuperMix-UDG (Invitrogen). In accordance with the manufacturer’s instructions, a 20-μL PCR reaction mixture contained 0.5 μL of RT product, 10 μL of 2× SYBR Green Mix, 0.4 μL of ROX, 0.8 μL of 10 μM primer mix, and 8.3 μL of nuclease-free water. The reaction protocol was: 95°C for 2 min, and then 40 amplification cycles of 95°C for 15 s, and 60°C for 30 s.

All reactions were run in triplicate. To account for possible differences in the amount of starting RNA, miRNA expressions were normalized to small nuclear RNA RNU6B [21,22]. RT-qPCR data were represented by the cycle threshold (Ct) value. The relative expression level (i.e., fold change) for each miRNA was calculated using the comparative cycle threshold 2^-ΔΔCt^ method [19].

### Target prediction and function analysis

We used the database miRFocus (http://mirfocus.org/) to predict potential human miRNA target genes. The website describes miRFocus as a human miRNA information database, and is an open-source web tool developed for rapid analysis of miRNAs. It also provides comprehensive information concerning human miRNAs, including not only miRNA annotations but also miRNA and target gene interactions, correlations between miRNAs and diseases and signaling pathways, and more. The miRFocus provides a full gene description and functional analysis for each target gene by combining the predicted target genes from other databases (TargetScan, miRanda, PicTar, MirTarget and microT). In this study, only those genes that were predicted by two or more databases were considered candidates; the greater the number of databases that predicted that a given gene would be a target, the more likely the miRNA-mRNA interaction would be relevant [23]. The miRFocus program also identifies miRNA-enriched pathways, incorporating those from the Kyoto Encyclopedia of Genes and Genomes (KEGG), Biocarta, and Gene Ontology (GO) databases, with Fisher’s exact test.

### Statistical analyses

All data are presented as mean ± standard deviation and analyzed with the paired *t*-test. Spearman’s correlation coefficients were used to examine the association between validated miRNAs and left atrial size. *P* < 0.05 was considered statistically significant.

## Results

### Clinical characteristics of the NSR and AF patients

There were no significant differences in terms of age, gender, or NYHA functional classification between the NSR and AF groups. Preoperative color Doppler echocardiography showed that the size of the left atria of AF patients was significantly greater than that of NSR patients as previously reported [24], while there were no differences in the left ventricular end-diastolic diameter or ejection fraction between the two groups of patients (Table 1).

**Table 1:** Clinical characteristics of the NSR and AF patients (n = 6, each)

	**NSR (n = 6)**	**AF (n = 6)**
Age (years)	47.51 ± 8.36	49.42 ± 11.87
Gender (male/female)	3/3	2/4
LA size (mm)	38.31 ± 5.32	56.35 ± 6.08*
LVEDD (mm)	42.35 ± 4.43	44.13 ± 5.17
LVEF (%)	62.54 ± 5.16	60.73 ± 4.04
NYHA classification	II (3/6)/III(3/6)	II (2/6)/III(4/6)

*P < 0.05 with respect to NSR patients.

### miRNA expression profiles of LAA tissue from MS patients with and without AF

**Figure 1:**
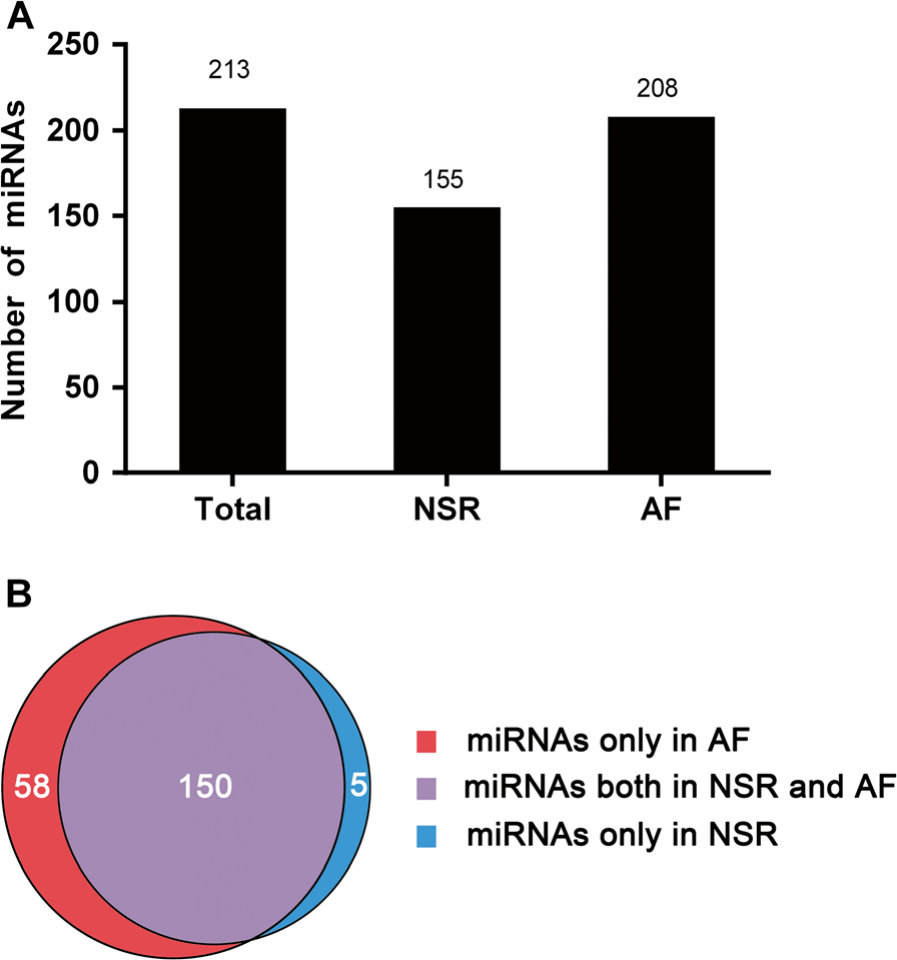
**MiRNAs detected in NSR and AF MS patients. (A)** A combined total of 213 miRNAs were detected. The LAAs of NSR patients expressed 155 miRNAs, while those with AF expressed 208 miRNAs. **(B)** Among these, 150 miRNAs were expressed in NSR and AF patients; 5 were expressed only in the NSR and 58 only in the AF.

Of the 1898 human miRNAs analyzed, a total 213 miRNAs were detected; NSR patients expressed 155 miRNAs, while the AF patients expressed 208 miRNAs (Figure 1A). Among these, 150 miRNAs were common to the patients of both groups. 5 miRNAs were detected only in those with NSR, and 58 miRNAs were detected only in those with AF (Figure 1B).

**Figure 2:**
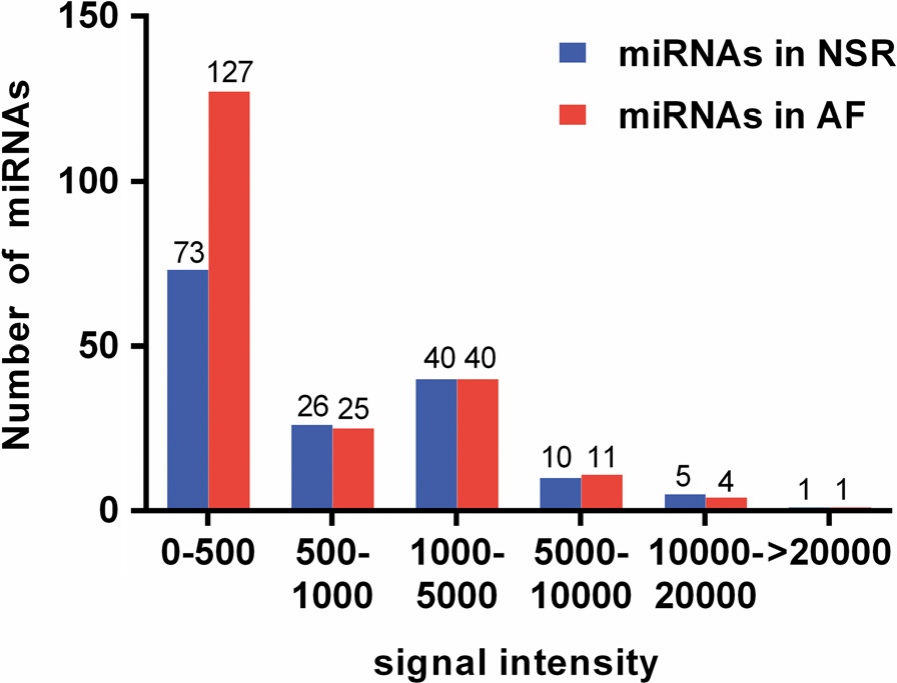
**Signal distribution of all detected miRNAs in MS patients, shown by microarray assay.** The signal intensities of most of these miRNAs were low (0–500 units), with only 11 miRNAs showing intensities greater than 10000 units.

However, the expression levels of most of the detected miRNAs were low, which is evident by their low signal intensities (less than 500 units). Of the 155 miRNAs detected in the NSR group, 73 emitted signals <500 units, while only 16 were >5000 units. On the other hand, of the 208 miRNAs detected in patients with AF, the signal intensities of 127 were <500 units, while only 16 were >5000 units (Figure 2). The signal intensities of the 5 miRNAs detected only in NSR, and the 58 miRNAs detected only in AF, were all <200 units and not high enough to consider them as differentially expressed between NSR and AF. Hence these were not considered here for further analysis.

### Differences in miRNA expression profiles of LAA tissues from MS patients with and without AF

Differences existed in the expression levels of the 150 miRNAs detected in both the NSR and AF groups (Table 2). Statistical analysis showed that 22 of these miRNAs (15%) were significantly dysregulated in the AF group relative to the NSR: 10 miRNAs (45%) were upregulated, while 12 (55%) were downregulated (*P* < 0.05).

**Table 2:** MiRNAs differentially expressed in LAA tissues between MS patients with AF or NSR

**miRNAs**	**NSR signal**	**AF signal**	**Log**_**2**_^**(AF/NSR)**^	***P*-value**
Upregulated*				
hsa-miR-3613-3p	294.01	3062.72	3.38	0.011
hsa-miR-3196	1383.37	2477.49	0.84	0.013
hsa-miR-3178	185.46	374.05	1.01	0.020
hsa-miR-466	1183.25	3342.81	1.50	0.023
hsa-miR-574-3p	4477.78	13515.75	1.59	0.024
hsa-miR-4492	334.39	834.17	1.32	0.027
hsa-miR-4707-5p	386.91	857.24	1.15	0.029
hsa-miR-15b-5p	382.60	723.26	0.92	0.030
hsa-miR-21-5p	442.29	819.40	0.89	0.047
hsa-miR-4497	4574.24	6545.73	0.52	0.047
Downregulated*				
hsa-miR-26a-5p	9701.87	4313.64	-1.17	0.007
hsa-miR-1	12740.13	3079.47	-2.05	0.019
hsa-miR-195-5p	611.70	384.70	-0.67	0.022
hsa-miR-26b-5p	794.41	198.33	-2.00	0.023
hsa-miR-5100	775.16	362.75	-1.10	0.029
hsa-miR-29a-3p	589.33	512.76	-0.20	0.029
hsa-miR-24-3p	3269.76	2405.77	-0.44	0.031
hsa-miR-361-5p	652.46	259.00	-1.33	0.035
hsa-miR-151a-5p	693.52	302.25	-1.20	0.039
hsa-miR-4454	1262.00	488.07	-1.37	0.039
hsa-miR-720	951.72	618.64	-0.62	0.047
hsa-let-7 g-5p	4801.17	3524.47	-0.45	0.047

*Expression levels of miRNAs are described as upregulated or downregulated in AF relative to those in NSR.

**Figure 3:**
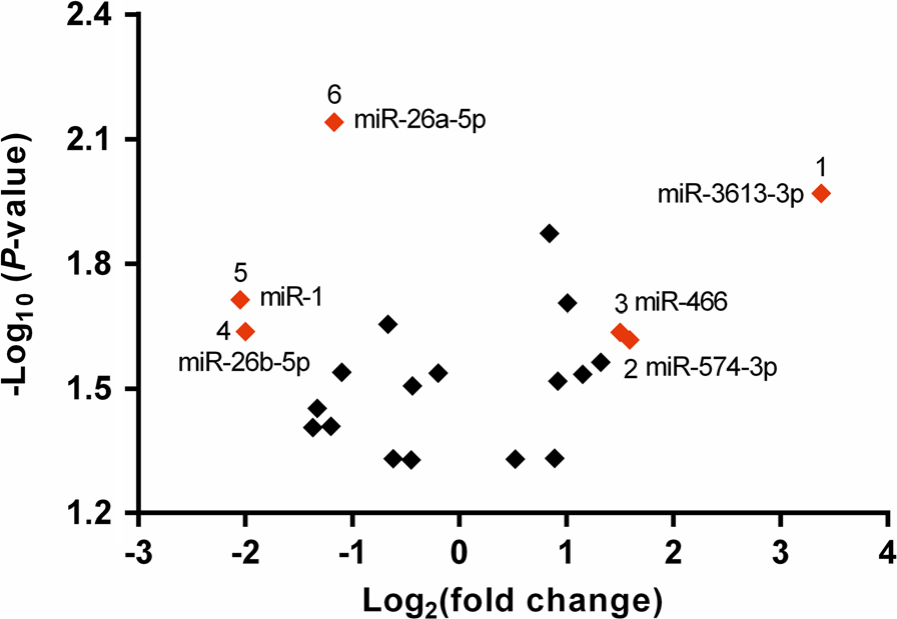
**Volcano plot of 22 miRNAs significantly dysregulated in LAA tissues.** The volcano plot shows the distribution of these miRNAs according to their *P*-value versus fold change. Those with the highest fold change in their expression (|Log_2_^(fold change)^| ≥ 1.5) have been labeled in red. Although the fold change of miR-26a-5p (|log_2_^(fold change)^| = 1.17) does not meet the criteria, its *P*-value is <0.01 (i.e., –Log_10_^(*P*-value)^ > 2), and therefore is also labeled red.

**Figure 4:**
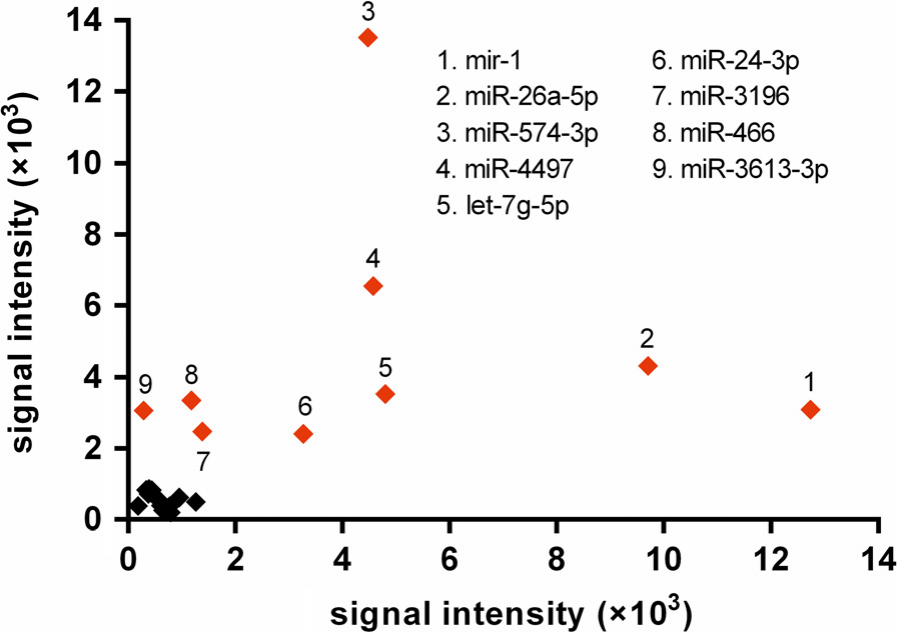
**Comparison of signal intensities of 22 miRNAs significantly dysregulated in LAA tissues.** The signal intensities of miRNAs expressed in the NSR group (x-axis) and AF group (y-axis) were compared. The miRNAs with the higher signal intensities (>2000 units) either on the x-axis or y-axis were labeled red.

Most of the miRNAs selected for further analysis via RT-qPCR had a fold change that satisfied the equation |log_2_^(fold change)^| ≥ 1.5(Figure 3), and at least one group had a signal intensity >2000 units (Figure 4). Although the fold change of hsa-miR-26a-5p did not meet this criteria (|log_2_^(fold change)^| = 1.17), its *P* -value was <0.01, and thus it was included in our selection. Finally, we selected 5 miRNAs for further analysis: 3 were upregulated in the AF group relative to the NSR (hsa-miR-466, hsa-miR-574-3p, and hsa-miR-3613-3p), and 2 were downregulated (hsa-miR-1 and hsa-miR-26a-5p).

### Validation of the miRNA microarray data with RT-qPCR

**Figure 5:**
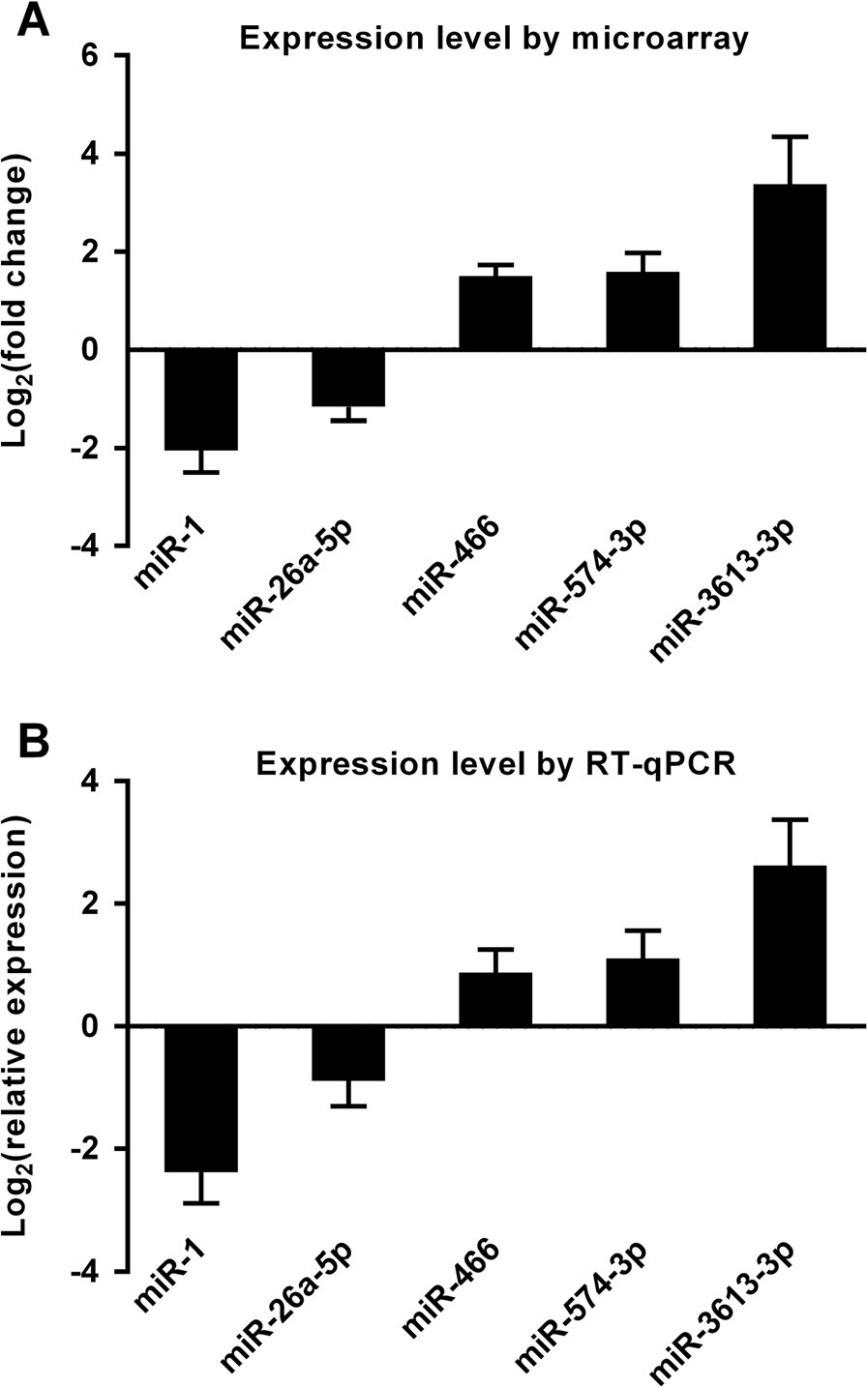
**Validation of miRNA microarray data of selected miRNAs by RT-qPCR. RT-qPCR was performed on 5 differentially expressed miRNAs (3 upregulated: miR-466, miR-574-3p, miR-3613-3p; and 2 downregulated: miR-1, miR-26a-5p) to confirm the microarray data. (A)** Illustration of microarray results. Expression levels of the 5 miRNAs in the AF group based on fold changes of signal intensity in the microarray relative to the NSR group. The log_2_^(fold change)^ values are plotted on the y-axis. **(B)** Illustration of RT-qPCR results. Using RT-qPCR, the expression levels of each miRNA in the AF group relative to the NRS group was estimated by the comparative cycle threshold 2^-ΔΔCt^ method. The log_2_^(relative expression)^ values are plotted on the y-axis. There is a significant difference for each miRNA, consistent with the microarray result.

To validate the data obtained from the miRNA microarray, RT-qPCR was performed on 5 selected miRNAs, and the results were compared with the microarray (Figure 5, Additional file 3). According to the RT-qPCR data, hsa-miR-466, hsa-miR-574-3p, and hsa-miR-3613-3p were upregulated in the LAAs of the AF group relative to the NSR, while hsa-miR-1 and hsa-miR-26a-5p were downregulated. These data are comparable with our microarray data and thus validate the results from the miRNA microarray for these miRNAs.

### Association between LA size and changes in miRNA expression

We investigated whether the changes observed in the expression levels of the 5 validated miRNAs between the LAA tissues of NSR and AF patients correlated with LA size.

**Figure 6:**
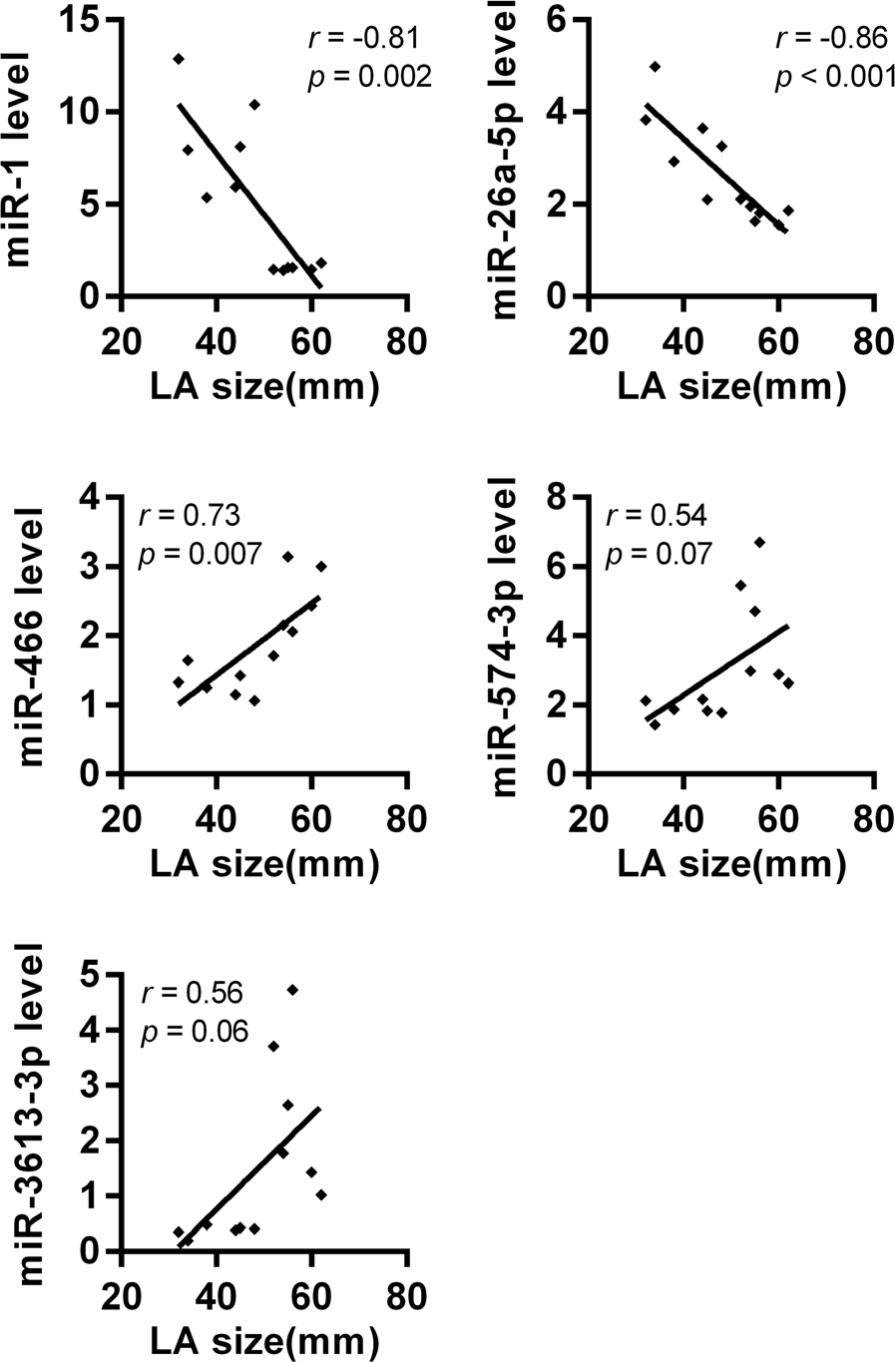
**Correlations between LA size and the relative expression levels of selected miRNAs.** Spearman’s correlation analysis showed a positive correlation between the level of expression of miR-466 and LA size, and a negative correlation between the level of expression of miR-1 and miR-26a-5p with LA size. The expression levels of miR-574-3p and miR-3613-3p were not significantly correlated with LA size.

Spearman’s correlation analysis showed a positive correlation between the level of expression of miR-466 in LAA and LA size (*r* = 0.73; *P* = 0.007). Moreover, there was a significantly negative correlation between the levels of expression of miR-1 and miR-26a-5p in LAAs and LA size (*r* = –0.81; *P* = 0.002 and *r* = –0.86; *P* < 0.001, respectively). However, although the expression levels of miR-574-3p and miR-3613-3p in LAAs significantly differed between NSR and AF patients, they were not significantly correlated with LA size (*r* = 0.54; *P* = 0.07 and *r* = 0.56; *P* = 0.06, respectively; Figure 6).

### Prediction of putative target genes and pathways of the differentially expressed miRNAs

To determine the probable biological function of the differentially expressed miRNAs, we predicted the putative targets and pathways of 5 validated miRNAs (hsa-miR-1, hsa-miR-26a-5p, hsa-miR-466, hsa-miR-574-3p, and hsa-miR-3613-3p) using the miRFocus database.

Numerous putative target genes and pathways were identified for the 5 miRNAs. The miRNAs hsa-miR-1 and hsa-miR-26a-5p were predicted by 5 target prediction databases; hsa-miR-574-3p was predicted from 4 target prediction databases, and hsa-miR-466 and hsa-miR-3613-3P were predicted from 2 target prediction databases (Table 3).

**Table 3:** Prediction of putative target genes and pathways of selected miRNAs

**miRNAs**	**Target gene**	**GO term**	**KEGG pathways**	**Target prediction database**
miR-1	613	41	2	miRanda, MirTarget, microT, PicTar, TargetScan
miR-26a-5p	591	16	2	miRanda, MirTarget, microT, PicTar, TargetScan
miR-466	312	50	2	MirTarget, TargetScan
miR-574-3p	31	42	3	MirTarget, microT, PicTar, TargetScan
miR-3613-3p	847	133	14	MirTarget, TargetScan

The biological function and potential functional pathways of each putative gene target were classified using the GO term and KEGG pathway (Tables 4 and 5). Since every gene is associated with many GO terms and KEGG pathways, the significant GO term and KEGG pathway for each miRNA were identified with Fisher’s exact test. Results of the target analysis indicated that the predicted potential genes have been linked to such important biological processes as the regulation of protein metabolism, transcription factor activity, cell division, and the transforming growth factor beta receptor (TGFBR) signaling pathway. These results suggest that these miRNAs have roles in human health and disease regulation. The pathway analysis also suggested that these miRNAs have important regulatory roles in different biological processes.

**Table 4:** Biological processes of the predicted miRNA targets

**GO process**	**Target genes**	***P*-value**
miR-1		
GO:0003723: RNA binding	DDX5 SFRS9 HNRNPU TRA2B CPEB1 SFRS1 HNRNPA3 HNRNPK SFRS3	6.57E-05
GO:0006397: mRNA processing	ISY1 DDX5 SFRS9 DHX15 HNRNPU CPEB1 SFRS1 HNRNPA3 HNRNPK SFRS3	5.69E-03
miR-26a-5p		
GO:0007179: transforming growth factor beta receptor signaling pathway	TGFBR2 MAP3K1 NLK	5.41E-04
GO:0051301: cell division	CCNE2 CCNE1 CDK6 CCND2	7.90E-03
miR-466		
GO:0003700: transcription factor activity	ARNT2 GLI3 SMAD2 RUNX1T1 TCF7L2 MECOM	4.56E-04
GO:0005515: protein binding	CCDC6 KIT CYCS EIF4E COL4A1 PTGS2 RUNX1T1 ULK2 MECOM RICTOR	6.19E-04
GO:0006468: protein amino acid phosphorylation	PRKAA2 RPS6KA3 SMAD2 ULK2 DAPK1	9.15E-04
GO:0046872: metal ion binding	CYCS GLI3 PTGS2 RUNX1T1 MECOM	6.58E-03
miR-574-3p		
GO:0031625: ubiquitin protein ligase binding	CUL2 ACVR1B	1.46E-03
GO:0051384: response to glucocorticoid stimulus	EP300 RXRA	3.87E-03
miR-3613-3p		
GO:0003700: transcription factor activity	NFATC3 NFAT5 EP300 RARA CREBBP RUNX1T1 RUNX1 ETS1 TCF7L2 NKX3-1 HDAC2 STAT3 CTNNB1 SMAD4	5.59E-13
GO:0004842: ubiquitin-protein ligase activity	UBE2J1 UBE2H UBE2Z BIRC6 MAP3K1 UBE2N UBE2D3 UBE2B NEDD4L LMO7 UBE2R2 HUWE1 UBE2E2 UBE2G1	5.61E-06
GO:0006355: regulation of transcription, DNA dependent	NFAT5 EP300 RARA CREBBP ABL1 RUNX1T1 RUNX1 NKX3-1 CREB1	1.65E-03
GO:0008134: transcription factor binding	PPARGC1A ETS1 TCF7L2 NKX3-1 HDAC2 PIM1 STAT3 CTNNB1	6.19E-06
GO:0008284: positive regulation of cell proliferation	FGFR2 FGFR1 ADAM17 INSR KIT BIRC6 HDAC2 IGF1R CCND1 KRAS	8.74E-04
GO:0046872: metal ion binding	PRKCI EP300 ACACB RARA CREBBP RUNX1T1 MID1 ADAM17 INSR PPP1CB PIM1 XIAP EGLN1 IGF1R LMO7 TRAF3	4.16E-04
GO:0051246: regulation of protein metabolic process	UBE2J1 UBE2H UBE2Z BIRC6 UBE2N UBE2D3 UBE2B UBE2R2 UBE2E2 UBE2G1	5.24E-05

**Table 5:** Pathway analysis of the selected miRNAs

**KEGG_Description**	**Target genes**	***P*-value**
miR-1		
hsa3040: Spliceosome	ISY1 DDX5 SFRS9 DHX15 HNRNPU TRA2B SFRS1 HNRNPA3 HNRNPK SFRS3	1.39E-03
hsa4320: Dorso-ventral axis formation	NOTCH2 EGFR NOTCH3 CPEB1 ETS1	3.86E-03
miR-26a-5p		
hsa4115: p53 signaling pathway	CCNE2 CCNE1 PTEN CDK6 CCND2 IGF1 ATM	1.98E-03
hsa4010: MAPK signaling pathway	TGFBR2 MYC PPP3R1 CACNA1C PPP3CB MAP3K1 NLK PAK2	8.40E-03
miR-466		
hsa4150: mTOR signaling pathway	PRKAA2 RPS6KA3 EIF4E ULK2 RICTOR	2.36E-03
hsa5200: Pathways in cancer	CCDC6 ARNT2 KIT CYCS COL4A1 GLI3 PTGS2 SMAD2 RUNX1T1 TGFB2 TCF7L2 DAPK1 MECOM	4.56E-03
miR-574-3p		
hsa5200: Pathways in cancer	CUL2 EP300 ACVR1B RXRA	3.75E-03
hsa5016: Huntington's disease	EP300 CLTC	4.38E-03
hsa5211: Renal cell carcinoma	CUL2 EP300	9.33E-03
miR-3613-3p		
hsa4520: Adherens junction	TCF7 EP300 CREBBP ACTN4 FGFR1 PTPRJ INSR TCF7L2 ACVR1C IGF1R CTNNB1 CTNND1 WASL LMO7 SMAD4	5.19E-06
hsa4310: Wnt signaling pathway	NFATC3 TCF7 NFAT5 EP300 CREBBP AXIN2 GSK3B PPP3CA VANGL2 TCF7L2 PPP2R5A WNT5A CCND2 PPP3R1 CCND1 CTNNB1 CSNK1A1 CSNK1E WNT4 SMAD4	9.31E-05
hsa4720: Long-term potentiation	EP300 CREBBP PPP1R12A PPP3CA PPP1CB PPP3R1 RPS6KA2 RAP1A GRIA1 KRAS	6.75E-04
hsa4330: Notch signaling pathway	JAG1 EP300 MAML3 CREBBP ADAM17 DLL1 NUMBL HDAC2	2.88E-03
hsa4910: Insulin signaling pathway	PRKCI PPP1R3B PPARGC1A ACACB GSK3B PRKAA1 INSR SOCS3 IRS1 EIF4E PPP1CB KRAS IRS2	6.80E-03
hsa4920: Adipocytokine signaling pathway	PPARGC1A ACACB PRKAA1 SOCS3 IRS1 STAT3 ACSL4 ACSL6 IRS2	8.20E-03

## Discussion

More and more studies indicate that specific alterations in miRNA expression profiles are associated with specific disease pathophysiologies [8,9,19]. Xiao et al. [16] were the first to report miRNA alterations in the RA associated with AF in MS patients; 28 miRNAs were differentially expressed between MS patients with AF and those in NSR. However, miRNA changes due to AF in the LA of MS patients are still unknown. The present study is the first to create and compare miRNA profiles of the LA of MS patients with AF and those without AF. We found that in the LA of MS patients, 22 miRNAs were differentially expressed between those with AF and those in NSR.

The results of our study and that of Xiao et al. [16] were completely different, except for miR-26b. After eliminating the influences of the miRNA microarray technologies used in the two studies, we conclude that these differences, at least in part, may reflect different mechanisms involved in AF between the LA and RA. In MS patients, electrical remodeling of both the left and right atria [14] is intrinsic to the initiation, development, and maintenance of AF [25], and morphological differences have also been demonstrated between the two atria [26]. Thus, it is not surprising that AF alters the miRNA expression profiles of the LA of MS patients, and that these alterations may differ from those of the RA. These differences may reflect different mechanisms involved in AF between LA and RA. Therefore, investigations into the differences in miRNA expression profiles associated with AF in MS patients should focus not only on the RA but also on the LA.

Cooley et al. [17] investigated the differences in miRNA expression profiles in LAA tissues from valvular heart disease patients, and found no detectable differences between patients with AF and those with NSR, a lack that these researchers attributed partially to problems with tissue availability. However, Girmatsion et al. [27] reported that miR-1 was downregulated in human LAA tissue from AF patients (relative to patients without AF who also underwent mitral valve repair or bypass grafting), which is consistent with our finding that miR-1 was downregulated in human LAA tissues in MS patients with AF. Unfortunately, Girmatsion et al.’s investigation did not utilize miRNA expression profiles. Recently, Luo et al. [28] found that miR-26 family members were significantly downregulated (>50%) in LAAs from a canine AF model (miR-26a) and in right atrial appendages from AF patients (miR-26a and miR-26b). This suggests the possible involvement of these miRNAs in AF pathophysiology, and is consistent with our finding that miR-26a-5p was downregulated in LAA tissues from MS patients with AF compared with those who remained in NSR.

Lu et al. [10] found that levels of miR-328 were elevated 3.5- to 3.9-fold in LAAs from dogs with model AF and in right atrial appendages from AF patients, detected by both microarray and RT-qPCR. However, in the present study we found that the expressions of miR-328 were very low in both the NSR and AF group and were not significantly different between the two groups. This contradictory finding may reflect differences in the species (dogs in Lu et al., humans in the present study) and in tissues that were sampled (right atrial appendages from AF patients in Lu et al., LAAs from AF patients in the current study) and the heterogeneity of human myocardial samples [29].

Studies have shown that miRNAs may be involved directly or indirectly in AF by modulating atrial electrical remodeling (miR-1, miR-26, miR-328) [10,27,28] or structural remodeling (miR-30, miR-133, mir-590) [7,30]. One study showed that miR-1 overexpression slowed conduction and depolarized the cytoplasmic membrane by post-transcriptionally repressing *KCNJ2* (potassium inwardly-rectifying channel, subfamily J, member 2; which encodes the K^+^ channel subunit Kir2.1) and *GJA1* (gap junction protein, alpha 1, 43 kDa; which encodes connexin 43), and this likely accounts at least in part for its arrhythmogenic potential [31]. Another study indicated that miR-1 levels are greatly reduced in human AF, possibly contributing to upregulation of Kir2.1 subunits, leading to increased cardiac inward-rectifier potassium current *I*_*K1*_[27]. A recent study identified miR-26 as a potentially important regulator of *KCNJ2* gene expression and, via I_K1_, a determinant of AF susceptibility [28]. In addition, it also identified miR-26 as a potential mediator of the electrophysiological effects of Ca^2+^-dependent NFAT (nuclear factor of activated T cells) signaling, believed to be important in the perpetuation of AF.

Previously, the miR-466, miR-574, and miR-3613 have not been described as participating in cardiovascular pathology. The current study found that these miRNAs are potentially involved in several important biological processes and functional pathways associated with AF (e.g., mTOR, Wnt, and Notch signaling), based on the predictions of putative target genes and pathways determined via miRFocus. Our results may implicate these miRNAs in the pathogenesis of AF.

In MS, the association between LA size and AF is well established and LA dilatation is considered both a cause and consequence of AF [13]. Our study found that the expression levels of three validated miRNAs (miR-1, miR-26a-5p, miR-466) correlated with LA size, while those of two others (miR-574-3p, miR-3613-3p) did not. This discrepancy is probably due to the multifactorial nature of AF in MS. For example, it is likely that persistent rheumatic inflammation and LA fibrosis also contribute to the etiology of AF in MS, as well as LA size and hypertension [13].

The main limitation of this study was the small number of patients included. This was due, in part, to the difficulty of finding MS patients with NSR. In addition, because this study was performed with native human tissues, we could not conduct experiments to modulate miRNA levels. Accordingly, the evidence presented here is indirect. Furthermore, the exact targets and pathways by which alterations in miRNAs cause AF in MS patients remain elusive and deserve further investigation [16]. Finally, the patients in this study were a specific cohort with preserved systolic left ventricular function and little comorbidity; they were undergoing mitral valve replacement surgery. Thus, changes identified in this population may not be representative of other cohort populations [27].

## Conclusions

This study shows that AF alters the miRNA expression profiles in LA from MS patients. These findings may be useful for the biological understanding of AF in MS patients and provide potential therapeutic targets for AF [32].

## Abbreviations

AF: Atrial fibrillation; CV: Coefficient of variation; GO: Gene Ontology; KEGG: Kyoto Encyclopedia of Genes and Genomes; LA: Left atrial; LAA: Left atrial appendage; miRNA: Microrna; MS: Mitral stenosis; NSR: Normal sinus rhythm; NYHA: New York Heart Association; RT-qPCR: Reverse-transcription quantitative PCR; TGFBR: Transforming growth factor beta receptor.

## Competing interests

The authors declare that they have no competing interests.

## Authors’ contributions

HL performed the molecular studies, participated in the sequence alignment, and drafted the manuscript. GXC, MYL, HQ, JR, and JPY participated in open heart surgery and collected clinical samples. HL and ZKW participated in the design of the study and performed the statistical analyses. ZKW and GXC conceived the study, participated in its design and coordination, and helped to draft the manuscript. HL and GXC contributed equally to this article. All authors read and approved the final manuscript.

## Pre-publication history

The pre-publication history for this paper can be accessed here:

http://www.biomedcentral.com/1471-2261/14/10/prepub

## Supplementary Material

Additional file 1 Raw signal values for all miRNAs in microarray. Click here for file

Additional file 2 Primers for RT-qPCR of miRNA and RNU6B. Click here for file

Additional file 3 RT-qPCR data. Click here for file
